# Prenatal 4-phenylbutyric acid administration during mid-gestation ameliorates ASD-like behaviors by reducing cortical endoplasmic reticulum stress in VPA-induced ICR and BTBR mouse models

**DOI:** 10.3934/Neuroscience.2026002

**Published:** 2026-01-20

**Authors:** Koichi Kawada, Seisuke Mimori, Nobuyuki Kuramoto, Kyosuke Uno

**Affiliations:** 1 Laboratory of Molecular Pharmacology, Faculty of Pharmaceutical Sciences, Setsunan University, 45–1 Nagaotoge-cho, Hirakata, Osaka 573–0101, Japan; 2 Department of Pharmacology, Faculty of Pharmaceutical Sciences, Chiba Institute of Science, 15–8 Shiomi-cho, Choshi, Chiba 288–0025, Japan; 3 Department of Clinical Medicine, Faculty of Pharmaceutical Sciences, Chiba Institute of Science, 15–8 Shiomi-cho, Choshi, Chiba 288–0025, Japan

**Keywords:** autism spectrum disorder, sociability, endoplasmic reticulum stress, glucose-regulated protein, 4-phenylbutyric acid

## Abstract

Autism spectrum disorder (ASD) is a neurodevelopmental condition characterized by deficits in social interaction and repetitive behaviors. Increasing evidence suggests that endoplasmic reticulum (ER) stress contributes to abnormal brain development in ASD; however, whether prenatal modulation of ER stress can prevent ASD-like phenotypes remains unclear. In this study, we investigated the effects of prenatal administration of the chemical chaperone 4-phenylbutyric acid (4-PBA) in two etiologically distinct ASD mouse models: valproic acid (VPA)-exposed Jcl:ICR (ICR) mice and BTBR *T^+^ Itpr3^tf^*/J (BTBR) mice. Social behaviors were evaluated using the three-chamber test, and repetitive behaviors were assessed by self-grooming duration. 4-PBA was administered to mid-gestation mice, and behavioral changes in the male offspring derived-two type ASD model (VPA and BTBR mice) were evaluated. 4-PBA reduced ER stress in the cerebral cortex of the offspring male VPA and BTBR mice. In particular, 4-PBA strongly inhibited the expression of 94-kDa glucose-regulated protein, an ER stress marker, in BTBR male mice. In addition, 4-PBA improved synaptic organizer expression and neuronal maturation, which are diminished in ASD, specifically in the cerebral cortex of VPA mice. Furthermore, 4-PBA improved social reciprocity, a behavior specific to ASD in male VPA and BTBR mice. In conclusion, ER stress during mid-pregnancy is strongly associated with the development of ASD symptoms. Furthermore, the reduction in ER stress by 4-PBA leads to the suppression of ASD symptoms. Autism spectrum disorder (ASD) is a neurodevelopmental disorder characterized by a lack of sociality, and the difficulty in forming social relationships often leads to various social problems. Prenatal administration of 4-PBA to mothers may reduce the risk of developing ASD, and we believe this could be a new approach to prevention.

## 1. Introduction

Autism spectrum disorder (ASD) is a neurodevelopmental disorder characterized by a lack of communication that becomes more pronounced during infancy and childhood. ASD is considered a congenital dysfunction of the central nervous system (CNS). However, the details of the pathogenesis of ASD remain unclear, as it is thought to be extremely complex because of the involvement of multiple factors. Several studies have identified genetic mutations in patients with ASD. One such group of mutations is related to synaptogenesis. The synaptogenic molecules neuroligin (NLGN), cell adhesion molecule 1 (CADM1), glutamatergic scaffolding proteins Shank3, and postsynaptic density protein-95 (PSD-95) are factors located in chromosomal regions with single nucleotide polymorphisms and copy number variations in individuals with ASD. They have been implicated in ASD development [1],[2]. Furthermore, these molecules are also required for the formation of glutamate-mediated excitatory and gamma-aminobutyric acid-mediated inhibitory synapses. Additionally, their association with synaptic plasticity related to amino acid metabolism in ASD development is being highlighted [3],[4].

The endoplasmic reticulum (ER) is a multifunctional organelle involved in protein folding and processing. When unfolded proteins accumulate in the ER lumen, they become stressed. Essentially, when the ER encounters abnormal accumulation of unfolded proteins during protein production, it initiates responses, such as translational repression of nascent proteins, known as the unfolded protein response (UPR), activation of UPR response genes by a transcription factor, and ER-associated degradation (ERAD) mechanisms. In ERAD, unfolded proteins are ubiquitinated and degraded by proteasomes, thereby providing protein quality control. ER stress causes neurodegenerative diseases, such as Alzheimer's and Parkinson's diseases [5],[6]. ER stress is increased by environmental factors, such as alcohol consumption, smoking, and a high-fat diet during pregnancy. Stress, such as oxidative stress, affects neurodevelopment, including neuronal proliferation, differentiation, and maturation [7],[8]. Additionally, environmental factors, such as diabetes, obesity, infections, alcohol, and some drugs, often induce ER stress load [9]. Dysregulation of synaptic organizers and cell-adhesion molecules during neurodevelopment is expected to increase the burden of protein folding and trafficking within the endoplasmic reticulum, thereby predisposing developing neurons to ER stress and unfolded protein response activation, which may contribute to ASD pathophysiology.

Previously, we reported that ASD was associated with ER stress and subsequent abnormalities in neuronal differentiation and neurite outgrowth [10]. Additionally, Shank3 and the glial-type glutamate transporters glutamate/aspartate transporter and glutamate transporter 1 contribute to ASD development [11],[12], indicating glutamate's significant involvement in ASD development. We have reported that ER stress induces abnormal neuronal differentiation and suppresses neuronal projection outgrowth [13]. In other words, we speculate that abnormal neuronal differentiation and neurite outgrowth via ER stress in the brain are likely to lead to ASD development. Therefore, we hypothesized that reducing ER stress during CNS development (i.e., after mid-pregnancy) would lead to the suppression of ASD onset.

Among pharmacological agents that modulate ER stress, 4-phenylbutyric acid (4-PBA) has been widely used as a chemical chaperone that enhances protein folding capacity and alleviates ER stress in various neurological and neurodevelopmental disease models. Importantly, 4-PBA is an FDA-approved therapeutic for urea cycle disorders and a low-molecular-weight chemical chaperone with a well-characterized in vivo safety profile. Systemic administration of 4-PBA has been shown to modulate ER stress and exert neuroprotective effects in multiple rodent models, including models of retinal degeneration and neurotoxicity [14],[15], supporting its use for investigating ER stress modulation in neurodevelopmental contexts. 4-PBA is metabolized by beta-oxidation of phenylacetate, which is conjugated with glutamine to form phenylacetylglutamine and excreted by the kidney [16],[17]. The first therapeutic use of 4-PBA was for the inhibition of platelet aggregation, as reported in the mid-1970s [18]. Since then, 4-PBA has been identified as an alternative mechanism for ammonia removal in urea cycle disorders [19]. The therapeutic potential of 4-PBA has significantly expanded, as it acts as a weak histone deacetylase (HDAC) inhibitor [20], an ER stress inhibitor [21], and influences mitochondrial and peroxisomal biosynthesis. The ER stress inhibitory activity induced by 4-PBA is attributed to its ability to inhibit protein aggregation. 4-PBA holds great promise as a therapeutic agent for CNS degenerative diseases because of its inhibitory effect on neuronal cell death. Additionally, 4-PBA inhibits the UPR and ERAD, which are elevated in diabetic models [22]. Above all, 4-PBA stands out as a therapeutic drug owing to its clinical availability, high safety profile, and abundant pharmacokinetic data.

Therefore, in this study, we used 4-PBA to alleviate ER stress in mid- and late pregnancy, and investigate its effect on ASD.

## 2. Materials and methods

### 2.1. Antibodies and chemicals

Valproic acid (VPA) and a rabbit polyclonal antibody against CADM1 (RRID: AB_10920756) were purchased from Sigma-Aldrich Co. (St. Louis, MO, USA). A mouse monoclonal antibody against KDEL was purchased from MEDICAL & BIOLOGICAL LABORATORIES CO. (Tokyo, Japan). Rabbit polyclonal antibodies against Shank3 (RRID: AB_2187584) and neuroligin1 (RRID: AB_10009677) was purchased from Novus Biologicals (Centennial, CO, USA). A rabbit polyclonal antibody against PSD-95 (RRID: AB_2799289) and a mouse monoclonal antibody against GAPDH (RRID: AB_2756824) were purchased from Cell Signaling Technology, Inc. (Danvers, MA, USA). A mouse monoclonal antibody against Tau (RRID: AB_1524475) was purchased from Abcam (Cambridge, UK). Mouse monoclonal antibodies against microtubule-associated protein-2 (MAP-2, RRID: AB_94856) were purchased from Millipore Corporation (Temecula, CA, USA). Horseradish peroxidase (HRP)-conjugated anti-mouse (RRID: AB_772210) and -rabbit IgG (RRID: AB_772206) antibodies were purchased from GE Healthcare (Buckinghamshire, UK). An Alexa Fluor® 488-conjugated anti-mouse IgG antibody (RRID: AB_2536161) was purchased from Thermo Fisher Scientific Inc. (Grand Island, NY, USA). Western Lightning Chemiluminescence Reagent Plus was obtained from PerkinElmer Life Science Products, Inc. (Boston, MA, USA).

### 2.2. Animals and treatment

**Figure 1.**
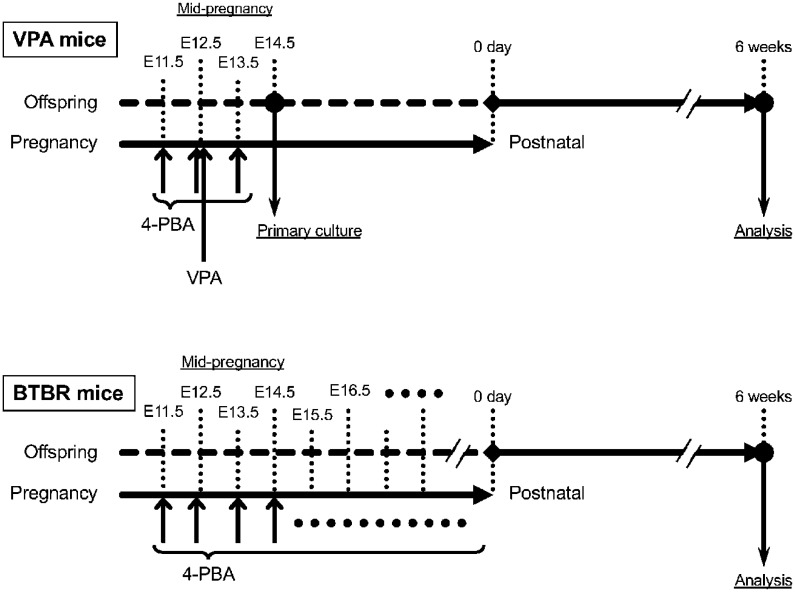
Time course. The figure shows the time course of the experiment using valproic acid and BTBR *T^+^ Itpr3^tf^*/J mice.

In this study, Jcl:ICR (ICR) mice (Japan SLC Corporation) were used for the valproic acid (VPA) model because of their well-documented sensitivity to prenatal VPA exposure and robust expression of ASD-like phenotypes. BTBR *T^+^ Itpr3^tf^*/J (BTBR) mice (The Jackson Laboratory) were employed as a complementary idiopathic/genetic ASD model. The use of these two models allowed us to examine whether ER stress represents a convergent pathological mechanism across etiologically distinct forms of ASD. ICR and BTBR mice were maintained at a temperature of 22°C under a 12/12-h light/dark cycle, with food and water provided ad libitum. In the preliminary experiment, almost equal numbers of male and female mice were used overall; however, sex differences in basic behaviors were not fully examined. It is well established that the male-to-female ratio in ASD patients is approximately 4–5 times higher in males than in females. Therefore, in experiments using sexed animals at 6 weeks of age, only male mice were used. Embryonic brain samples were collected as sex-mixed because sex determination was not performed at embryonic day (E) 14.5. In contrast, all behavioral experiments were conducted exclusively in male offspring, reflecting the higher prevalence of ASD in males. The use of sex-mixed embryonic samples may increase biological variability and is considered a limitation of this study. Additional experiments were performed when results were likely to differ, and the number of animals used was minimized whenever possible. Group assignments were randomized, and experimenters were blinded to mouse allocation during data acquisition and analysis. All experiments were conducted in accordance with the animal experimentation guidelines of Setsunan University and Chiba Institute of Science. Ethical approval was obtained from the Ethics Committees of Setsunan University (permit number: K25–16) and Chiba Institute of Science (permit numbers: 20–51, 20–52, 21–56, and 21–57) prior to the initiation of the study. As shown in Figure 1, VPA-induced ASD model mice of the ICR lineage were generated by intraperitoneal injection of valproic acid (500 mg/kg) into pregnant dams at gestational day 12.5. Offspring born to VPA-treated mothers were used as ASD model mice for experiments conducted either at E14.5 or at 6 weeks of age. Malformed offspring observed at E14.5 or at birth were excluded from the experiments. 4-PBA was administered intraperitoneally at a dose of 100 mg/kg/day to VPA mice from gestational day 11.5 to 13.5 to target ER stress during the critical window surrounding VPA exposure at mid-gestation. In contrast, BTBR mice were treated by 4-PBA from gestational day 11.5 until birth to modulate sustained prenatal ER stress associated with their genetic vulnerability. The dose of 4-PBA (100 mg/kg/day) was selected based on previous studies demonstrating effective reduction of ER stress and neuroprotective effects in rodent models without overt maternal or embryonic toxicity.

### 2.3. Primary culture

Primary cultured neurons were prepared from the cerebral cortex of embryonic day 14.5 (E14.5) in about 10 littermates of mixed sexes. Cells were grown in growth medium consisting of 64% Dulbecco's Modified Eagle Medium/F-12, 0.12% sodium bicarbonate, 0.01% penicillin-streptomycin solution, 16.5 mM glucose, 0.06 mM putrescine, 0.005 µg/mL sodium selenite, 0.02 µM progesterone, 0.8 µg/mL apo-transferrin, 0.25 mg/mL insulin, and 10 ng/mL epidermal growth factor at a density of 105 cells/mL and cultured for 4 days. The cultures were always maintained at 37°C in a 95% (v/v) humidified air atmosphere with 5% (v/v) carbon dioxide.

### 2.4. Immunoblot

The collected primary cultured cells were suspended in homogenizing buffer (including 10 mM Tris-HCl buffer (pH 7.6), 320 mM sucrose, 1 mM EDTA, 1 mM EGTA, 1 mM DTT, 1 mM sodium bata-glycerophosphate, 1 mM Na_3_VO_4_, 10 µg/mL aprotinin, 10 µg/mL leupeptin, and 1 mM PMSF) and then ultrasonically disrupted, after which protein was quantified and the cells were used as samples for the immunoblot. Cell lysates were boiled at 100°C for 10 min in 10 mM Tris-HCl buffer (pH 6.8) containing glycerol, sodium dodecyl sulphate, bromophenol blue, and 2-mercaptoethanol, and then stored at –80°C until use. Equal protein weight of the lysates was loaded onto a 5%–10% polyacrylamide gel for electrophoresis at a constant voltage of 100 V for 120 min at a temperature of 25°C. Subsequently, it was blotted onto a polyvinylidene fluoride membrane that had previously been treated with 100% methanol. After blocking with 5% skim milk dissolved in 20 mM Tris-HCl buffer (pH 7.5) containing 137 mM sodium chloride and 0.05% Tween 20 (TBST), the membranes were incubated with primary antibodies against KDEL (1:1000), Shank3 (1:1000), PSD-95 (1:1000), CADM1 (1:1000), NLGN1 (1:1000), and GAPDH (1:2000) for 2 h at room temperature. After washing with TBST, the membranes were incubated with horseradish peroxidase-conjugated secondary antibodies for 1 h at room temperature. Proteins reactive with the antibody were detected using Western Lightning Chemiluminescence Reagent Plus and then quantified using an LAS-3000 luminescence image analyzer (Fujifilm, Tokyo, Japan).

### 2.5. Immunocytochemistry

The cells were fixed with cold methanol for 15 min at 4°C and then blocked with Image-iT FX signal enhancer (Life Technologies) for 60 min. Subsequently, they were reacted with appropriately diluted primary antibodies against tau (1:200) and microtubule-associated protein-2 (MAP-2, 1:200) overnight at 4°C. The cells were then reacted with the corresponding secondary antibody, an anti-mouse IgG antibody conjugated to Alexa Fluor 488 (1:200). Subsequently, they were washed with 0.03% TBST containing Hoechst33342 at room temperature. After rinsing with TBST for 5 min, the cells were examined under an LSM 510 Meta confocal microscope (Carl Zeiss, Oberkochen, Germany). The neurite lengths of tau- and MAP-2-positive cells were measured in four different visual fields, with five cells randomly selected per visual field on each coverslip. The neurites were analyzed by observation with a 100x objective lens. The neurite length was averaged across the five cells in each visual field, and quantified using Cell Insight CX5 HCS Platform (Thermo Fisher Scientific Inc.).

### 2.6. Behavioral tests

Anxiety tests in the open-field box, social communication activities, and the three-chamber test were measured using SMART V3.0 (Bioresearch Center, Japan). The mice were acclimatized for 30 min in the trial room. For the anxiety test, the mice were tested in an open-field box (a grey circular plastic cage, 50 cm in diameter) for 15 min. All ambulatory movements in the center and periphery of the cage were recorded for further data analysis. The peripheral region was defined as 10 cm away from each side. Social communication was tested using an open field (50 cm long, 50 cm wide, and 30 cm high) divided into two compartments by transparent acrylic panels. The smaller compartment was 15 cm long, 50 cm wide, and 30 cm high. However, the larger compartment was 35 cm long, 50 cm wide, and 30 cm high. Prior to training, the animals were placed in the large compartment for 10 min with nothing in the field to acclimatize them to the field and allowed to move freely. Mice of the same strain, age, and sex but with no previous contact (novel mice) were placed in the smaller compartment, whereas the experimental mice were placed in the larger compartment for 600 s. The time that the experimental mice remained within 10 cm of the acrylic plate was recorded over the entire 600 s. The preferences for sociability and social novelty were assessed using a three-chamber test. The field was constructed in a laboratory using opaque polycarbonate. Each chamber was 20 cm long, 50 cm wide, and 30 cm high, with a 6-cm-wide opening to allow movement from the center to the adjacent chamber. Each columnar holder was 7 cm in diameter and was closed at the top and bottom. Each experimental mouse was placed in the central chamber of the three-chamber area and allowed to explore freely for 10 min in each of the three consecutive trials (habituation, sociability, and social novelty preference), all conducted on the same day. The interval between habituation and sociability was 10 min, and the interval between sociability and social novelty preference was 10 min. The mice used in this study were strain-, week-, and sex-matched, and housed in an environment where they had no previous contact with the test mice. During training (habituation), the experimental mice were placed in a center field and allowed to explore the three chambers (left, center, and right). In Trial 1 (social novelty), unfamiliar conspecific mice were placed in the “holder” of one of the two side chambers, with the other side chamber left empty. In Trial 2 (social novelty preferences), the stranger mouse from Trial 1 was left intact, whereas another stranger mouse of the same species (novel stranger mouse) was placed in the empty holder on the opposite side. The time spent in each chamber for 5 min from the start of each trial was measured (Trial 1 and 2).

### 2.7. Statistical analyses

All statistical tests were performed using the Statcel2 software. Data distribution was assessed for normality prior to statistical analysis. To compare two groups, we used an unpaired Student's t-test (data of neurite length), and for multiple comparisons, we used a one-way analysis of variance, followed by Bonferroni correction (data of immunoblot and behavior). Statistical significance was set at p ≤ 0.05. No data transformation was applied, and no statistical outliers were excluded. Sample sizes are indicated in each figure legend.

## 3. Results

### 3.1. 4-PBA reduces the increased endoplasmic reticulum stress load in the cerebral cortex of VPA mice

**Figure 2.**
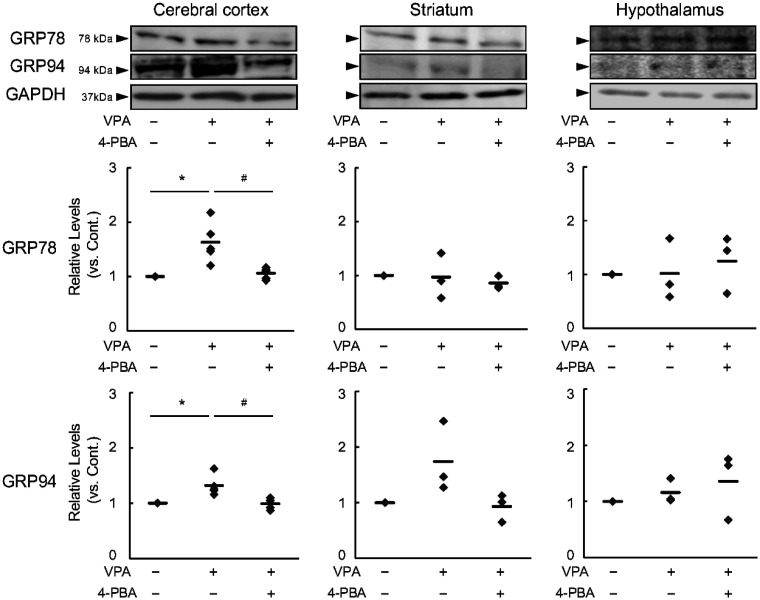
Effects of endoplasmic reticulum (ER) stress and 4-phenylbutyric acid (4-PBA) in valproic acid (VPA) mice. VPA mice were 6-week-old male offspring born to pregnant mice that received 500 mg/kg VPA intraperitoneally on gestation day 12.5. 4-PBA was administered to pregnant mice at 100 mg/kg/day on gestation days 11.5–13.5. 78-kDa glucose-regulated protein (GRP) and 94-kDa GRP were detected via immunoblot analysis using an anti-KDEL antibody. Data are presented as the mean and individual value of five (cerebral cortex, n = 5) and three (striatum and hypothalamus, n = 3) independent experiments. Statistical analysis was performed using one-way analysis of variance with Bonferroni correction for significant differences (*p < 0.05, compared with the control group; ^#^*p* < 0.05, compared with the VPA mouse group).

First, we examined the level of ER stress in the adult offspring. Findings showed that the levels of GRP78 and GRP94, which are markers of ER stress, were elevated in the cerebral cortex of 6-week-old VPA mice. However, these elevations were ameliorated by pretreatment with 4-PBA (Figure 2, left). In the striatum, GRP78 expression was not altered; however, GRP94 expression was increased by VPA treatment and decreased by 4-PBA pretreatment (Figure 2, middle). No significant changes in GRP78 or GRP94 expression were observed in the hypothalamus (Figure 2, right).

### 3.2. 4-PBA normalizes the expression of synaptic organizers in the cerebral cortex of adult mice induced with VPA

**Figure 3.**
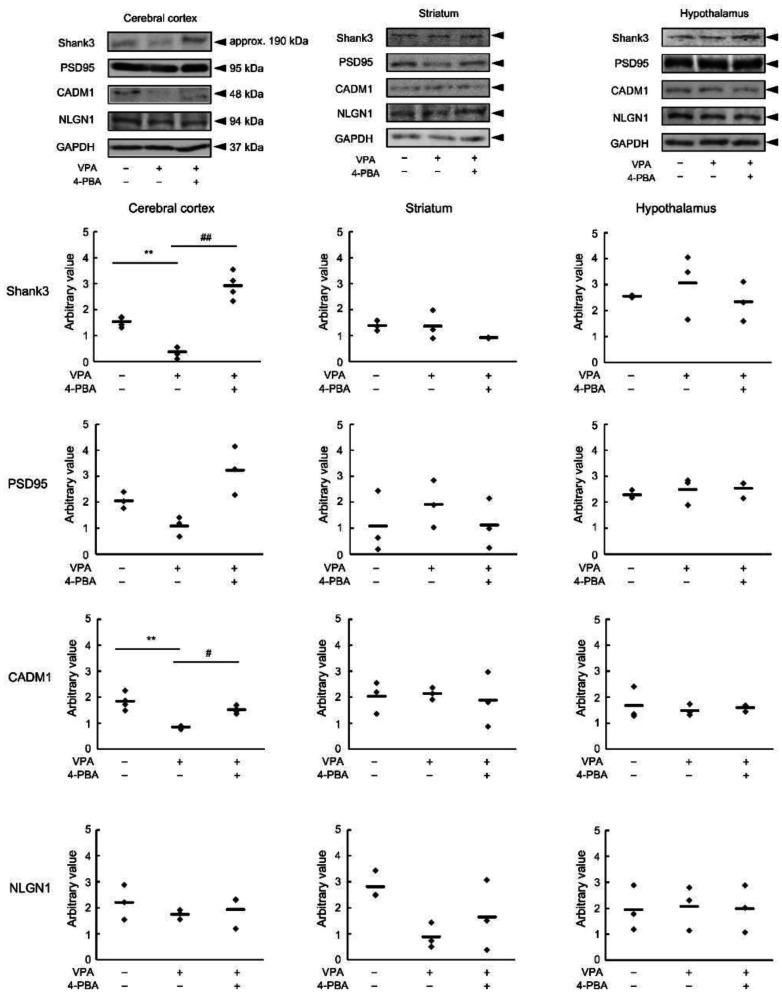
Effect of 4-phenylbutyric acid (4-PBA) on changes in synaptic organiser expression in valproic acid (VPA) mice. The expression of synaptic organisers (SH3 and multiple ankyrin repeat domains protein 3 [Shank3], postsynaptic density protein-95 [PSD95], cell adhesion molecule 1 [CADM1], and neuroligin-1 [NLGN1]) in the cerebral cortex, striatum, and hypothalamus of the VPA mice was determined via immunoblot analysis. The image shows the immunoblot results for Shank3, PSD95, CADM1, NLGN1, and glyceraldehyde-3-phosphate dehydrogenase (GAPDH) in the cerebral cortex of VPA mice. GAPDH corrected the expression of different synaptic organisers. The number of examples is given numerically in the graph. Data are presented as the mean and individual value of three (PSD95 and NLGN1 by the cerebral cortex, and all detection by the striatum and the hypothalamus, n = 3) and four (Shank3 and CADM1 by the cerebral cortex, n = 4) independent experiments. Statistical analysis was performed using one-way analysis of variance with Bonferroni correction for significant differences (**p < 0.01, compared with the control group; ^#^*p* < 0.05, ^##^*p* < 0.01, compared with the VPA(+)/4-PBA(-) mouse group).

In previous studies, we reported that ER stress affected the differentiation lineage of the nervous system [13]. In response to ER stress, cells sequentially activate three defense mechanisms: repression of translation of new proteins, repair of abnormal proteins by molecular chaperones, and ERAD via ubiquitination. We have previously reported that 4-PBA normalizes the neuronal differentiation pathway by suppressing the function of ERAD. In contrast, 4-PBA is a chemical chaperone that performs the same function as the molecular chaperone in the UPR. Based on the results shown in Figure 2, we hypothesized that the cerebral cortex of fetal VPA mice is under ER stress and that the use of 4-PBA, a chemical chaperone, affects the expression of proteins involved in ASD development. We found that Shank3 expression was suppressed in the cerebral cortex of the VPA mice, but this suppression was significantly ameliorated by 4-PBA (Figure 3, Shank3). In contrast, no significant changes were observed in the striatum or hypothalamus. CADM1 expression was suppressed in the cerebral cortex of VPA mice, and this suppression was significantly ameliorated by 4-PBA (Figure 3, CADM1). For PSD-95 and NLGN1, no significant changes were observed in either region (Figure 3, PSD-95 and NLGN1).

### 3.3. 4-PBA promotes axon outgrowth in neurons derived from the cerebral cortex of VPA mice

**Figure 4.**
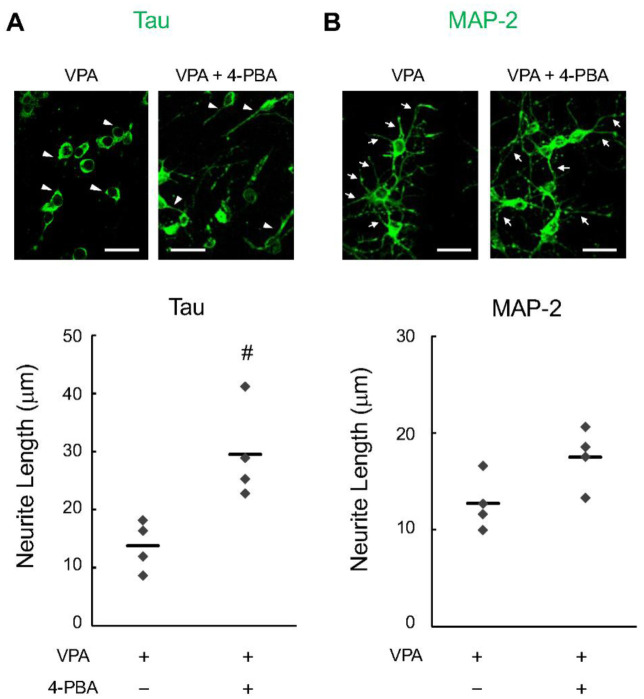
Effect of 4-phenylbutyric acid (4-PBA) on neurite outgrowth derived from valproic acid (VPA) mice. Neurons from VPA mice were isolated from the cerebral cortex of E14.5. Neurons were then cultured for 4 days and immunostained via immunocytochemistry: Tau (A) is a marker for neuronal axons, and microtubule-associated protein-2 (B) is a marker for neuronal dendrites (green). The measurements were made by averaging the lengths of neurite (green) from four randomly obtained visual fields. Arrowheads show the axons of Tau-positive cells, and arrows show the dendrite of MAP2-positive cells. Data are presented as the mean and individual value of four independent experiments (n = 4). Statistical analysis was performed using Student's t-test for significant differences (^#^*p* < 0.05, compared with the VPA mouse group). Scale bar: 20 µm.

Previous results have indicated that 4-PBA increases the expression of synaptic organizers in some neurons [23]. However, synaptogenesis and neurite outgrowth are considered to form independently of each other [24]. Therefore, we immunocytologically analyzed the effects of 4-PBA on neurite maturation (i.e., neurite outgrowth). The expression of tau (Figure 4A) and MAP-2 (Figure 4B), marker proteins of neuronal axons and dendrites, respectively, revealed that the axon length of tau-positive cells was significantly increased in the 4-PBA group (Figure 4A). In contrast, the dendritic length of the MAP-2-positive cells was not affected by 4-PBA treatment (Figure 4B).

### 3.4. Pretreatment with 4-PBA ameliorates abnormal behavior in the adult VPA offspring

**Figure 5.**
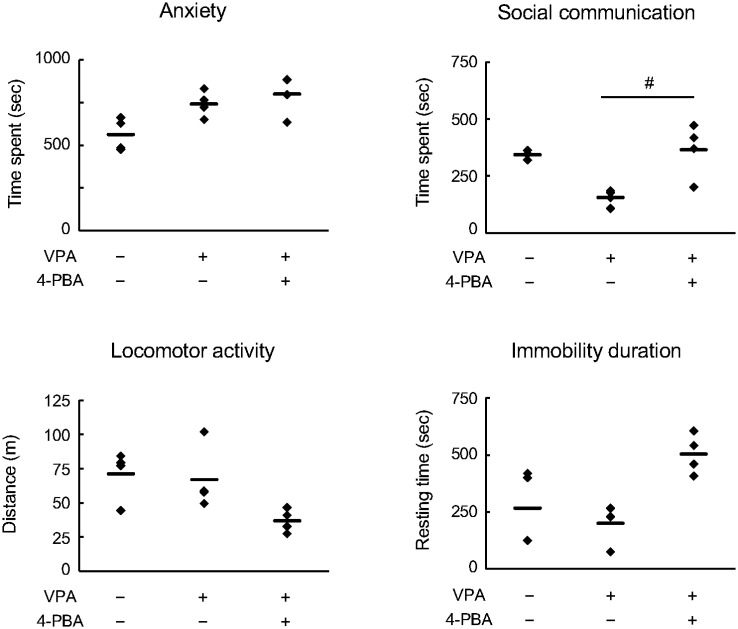
Effects of 4-phenylbutyric acid (4-PBA) on autism spectrum disorder-like abnormal behaviours in valproic acid (VPA) mice. We examined anxiety behaviours and social communication in 6-week-old VPA mice. Anxiety was assessed using a circular open field measuring 50 cm in diameter. The amount of time spent by the mice within 10 cm from the wall of the field was measured. Social communication was measured using a square open field of 50 cm on each side, and the time spent in the vicinity of an area within 10 cm of a stranger facing the mouse in one direction within the field was measured. A clear acrylic plate was used to prevent contact with the stranger mouse. Locomotor activity was quantified as the total distance traversed by the mouse, whereas immobility duration was defined as the time during which the mouse exhibited no ambulatory movement. Both measurements were performed within 900 s. The number of examples is given numerically in the graph. Data are presented as the mean and individual value of four independent experiments (n = 4). Statistical analysis was performed using one-way analysis of variance with Bonferroni correction for significant differences (^#^*p* < 0.05, compared with the VPA mouse group).

In mid-pregnancy, 4-PBA effectively ameliorated abnormal synaptogenesis and neurite outgrowth, which is typical of ASD. As in previous experimental designs, we investigated how pretreatment of maternal mice with 4-PBA alone affects behavioral abnormalities in their offspring. We then analyzed anxiety and social communication in adult offspring at 6 weeks of age. The results indicated that the VPA offspring exhibited mild anxiety behavior by spending more time at the edge of the circular field compared with those of untreated mice. In contrast, the combined VPA and 4-PBA group showed no change in the time spent at the edge compared with the VPA group (Figure 5, anxiety). In terms of social behavior, the VPA mice exhibited a decreasing trend in the time spent approaching the acrylic plate compared with the untreated group, whereas the combined VPA and 4-PBA group spent more time approaching the plate than did the VPA group (Figure 5, social communication). Additionally, in the combined VPA and 4-PBA group, locomotor activity indicated a declining trend, whereas immobility time exhibited an increasing trend compared with the untreated group (Figure 5, locomotor activity and immobility duration).

### 3.5. Pretreatment with 4-PBA suppresses the development of autism spectrum disorder-like behavioral abnormalities in BTBR offspring

**Figure 6.**
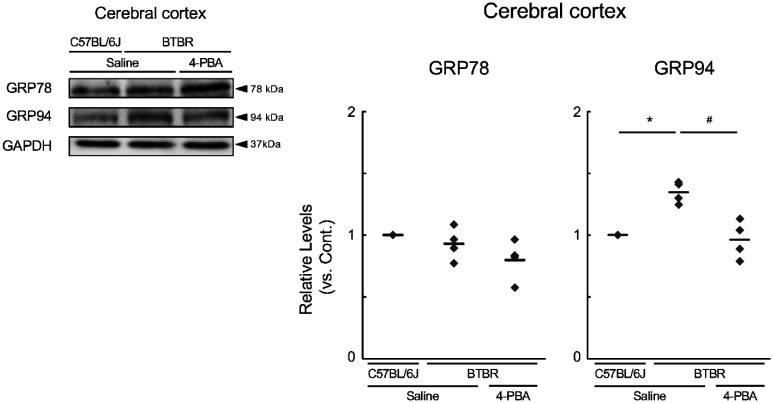
Effects of endoplasmic reticulum (ER) stress and 4-phenylbutyric acid (4-PBA) in BTBR *T^+^ Itpr3^tf^*/J (BTBR) mice. BTBR mice are a popular mouse model for autism spectrum disorder, and 6-week-old male offspring were used in this experiment. 4-PBA was administered to pregnant mice at 100 mg/kg/day from gestation day 11 until birth. 78-kDa glucose-regulated protein (GRP78) and 94-kDa GRP were detected via immunoblot analysis using an anti-KDEL antibody. Data are presented as the mean and individual value of four independent experiments (n = 4). Statistical analysis was performed using one-way analysis of variance, and significant differences were determined by Bonferroni correction (*p < 0.05, compared with the control group; ^#^*p* < 0.05, compared with the BTBR mouse group).

**Figure 7.**
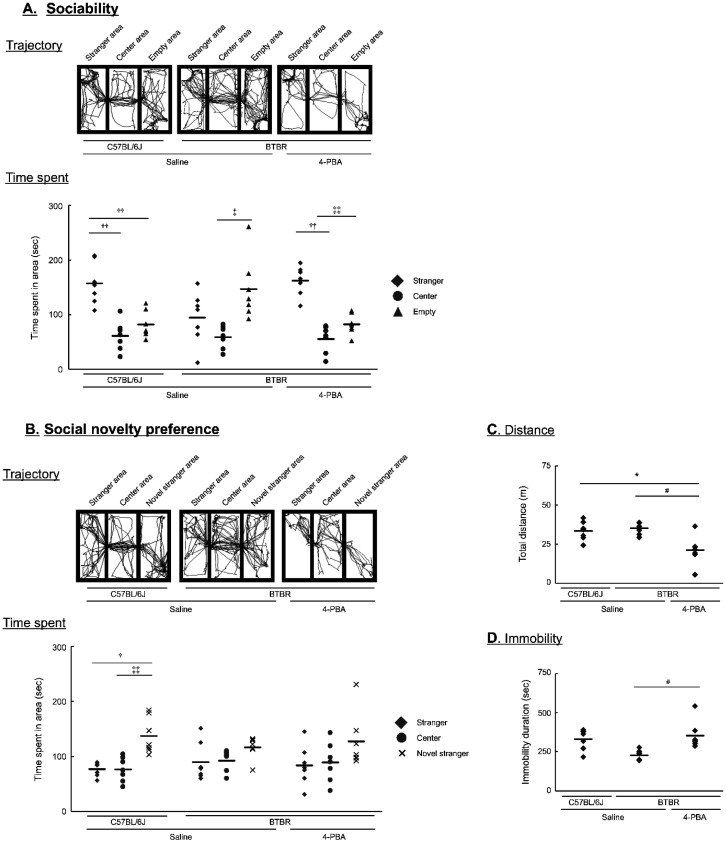
Effect of 4-phenylbutyric acid (4-PBA) on autism spectrum disorder-like abnormal behaviour in BTBR *T+ Itpr3^tf^*/J (BTBR) mice. (A and B) The sociability (A) and social novelty preferences (B) of 6-week-old BTBR mice were investigated. Behavioural trajectories of mice during behavioural analysis. Time spent in each of the three regions was measured. (C and D) Locomotor activity was quantified as the total distance traversed by the mouse, whereas immobility duration was defined as the time during which the mouse exhibited no ambulatory movement. The measurement time was 300 s for all analyses. Data are presented as the mean and individual value of seven independent experiments (n = 7). Statistical analysis was performed using one-way analysis of variance with Bonferroni correction for significant differences (*p < 0.05, compared with the control group; ^#^*p* < 0.05, compared with the BTBR mouse group; ^††^*p* < 0.01, ^†^*p* < 0.05, compared with the stranger area; ^‡‡^*p* < 0.01, ^‡^*p* < 0.05, compared with the centre area).

It is clear that 4-PBA improved social communication in VPA-treated offspring. To further investigate the ameliorative effects of 4-PBA on ASD-like behavior, we used BTBR mice, which are widely used as models of ASD, and investigated the effects of 4-PBA on ASD-like behavior using a three-chamber test. Various behavioral assessments were evaluated using the automated behavior analyzer SMART 3.0. Prior to this analysis, we checked for ER stress in BTBR mice at 6 weeks of age and found that GRP78 expression was unchanged between the control and BTBR mice (Figure 6). In contrast, GRP94 expression was higher in BTBR mice than that in control mice; however, this increase was alleviated by 4-PBA treatment (Figure 6). In this experiment, the total movement distance, average movement speed, and average immobility time of BTBR mice within the 5-min field did not differ between the treatment groups. In the social evaluation of the three-chamber test, C57BL/6J mice, used as the control group, stayed longer in the stranger mouse area than in the other two areas (Figure 7A). In contrast, BTBR mice spent more time in empty areas. However, treatment of BTBR mice with 4-PBA increased the time spent in stranger mouse areas, similar to the controls (Figure 7A). In the social novelty preference assessment, the C57BL/6J mice stayed longer in the novel stranger mouse area than in the other two areas. In contrast, BTBR mice spent equal time in all areas, similar to C57BL/6J mice in the 4-PBA-treated BTBR group (Figure 7B). Furthermore, the 4-PBA-treated BTBR mice exhibited lower locomotor activity compared with the control and BTBR groups (Figure 7C). Conversely, the immobility times in the 4-PBA-treated BTBR group was higher compared with that of the BTBR group (Figure 7D).

## 4. Discussion and conclusions

The administration of VPA at E12.5, rather than at E9.5 or E14.5, has been indicated to result in epigenetic dysregulation due to HDAC inhibition. This has been observed to induce apoptosis in the fetal neocortex and to inhibit cell proliferation in the basal ganglia primordia [25]. Thus, HDAC inhibition by VPA during this critical developmental window is considered an important contributor to the aberrant neurodevelopmental and behavioral phenotypes associated with prenatal VPA exposure. Although VPA mice exhibit ASD-like symptoms, the genetic factors underlying ASD are complex; therefore, in this study we also used BTBR mice to investigate the effect of 4-PBA in reducing ASD-like symptoms. Interestingly, it is known that the expression of ASD-like symptoms caused by VPA often occurs specifically in ICR mice, so we used ICR mice in this study. Additionally, HDAC inhibitors induce ER stress and modulate the unfolded protein response (UPR) [26]. During the embryonic period, embryonic cells are particularly susceptible to ER stress owing to the high number of newly generated cells and highly active metabolism [27]. Normally, the accumulation of unfolded proteins and increased ER stress activate pro-survival UPR signaling to maintain ER homeostasis. However, excessive ER stress during the embryonic period leads to abnormalities in neuronal differentiation pathways and in the expression of synaptic organizers [13]. Therefore, epigenetic abnormalities induced by VPA at E12.5 in regions related to neuronal differentiation or synaptic organizers may impose a long-lasting ER stress burden that persists into adulthood. On the other hand, when using embryonic primary cultures, sex determination is not possible, so we used sex-mixed mice.

In a previous study, we reported that ER stress during neurodevelopmental stages induced abnormal neuronal differentiation and inhibited neurite outgrowth [13]. In addition, postnatal administration of phenylbutyrate derivatives has been reported to ameliorate ASD-like phenotypes in related mouse models [28]. Based on these findings, we hypothesized that ER stress–mediated abnormalities in neuronal differentiation and neurite outgrowth contribute to the behavioral abnormalities observed in ASD. Accordingly, we focused on the inhibitory effects of 4-PBA on protein aggregation and ER stress reduction [29], and analyzed changes in synaptic organizer expression induced by 4-PBA in the brains of ASD model mice. First, analysis of ER stress in the VPA-induced ASD model confirmed that ER stress in the cerebral cortex remained elevated even in adulthood. Notably, increased ER stress, indicated by elevated levels of GRP78 and GRP94, was detected only in the cerebral cortex. In the striatum, only GRP94 levels were increased, whereas no increase in ER stress markers was observed in the hypothalamus. Importantly, all of these GRP increases were attenuated by prenatal 4-PBA pretreatment. The differential regulation of GRP78 and GRP94 among the cortex, striatum, and hypothalamus may reflect region-specific developmental timing and functional demands. In fact, the hypothalamus, striatum, and cerebral cortex form in that order during brain development. The hypothalamus forms around E9, while the cerebral cortex begins forming after E11 [30]–[32]. GRP78 functions as a general endoplasmic reticulum chaperone, while GRP94 is closely associated with neurodevelopmental processes, such as cell adhesion and synapse formation. These developmental gaps may explain the significant changes observed exclusively in the cortex after embryonic day 11. The question that arises is how GRP78 and GRP94 are differentially involved in ASD. Both GRP78 and GRP94 are molecular chaperones whose expression is induced by ER stress; however, GRP78 is ubiquitously expressed across species, including yeast, and primarily assists in protein folding and canonical ER stress responses [33]. In contrast, GRP94 is expressed only in multicellular organisms and plays critical roles in developmental processes and cell adhesion [33]. Therefore, sustained upregulation of GRP94 under ER stress conditions is likely to impair cell adhesion and synaptogenesis, thereby contributing to abnormal neural circuit formation associated with ASD-like phenotypes. Although the present findings suggest that suppression of GRP94 expression in the cerebral cortex and striatum may be associated with the amelioration of ASD-like behaviors by 4-PBA, direct causal evidence linking GRP94 modulation to behavioral rescue is currently lacking. Therefore, GRP94 should be regarded as a potential key mediator rather than a definitive determinant of ASD-like phenotypes. Moreover, ASD-related neurodevelopmental abnormalities are unlikely to be explained by a single ER stress component. Other branches of the UPR, including the IRE1-XBP1 and PERK-eIF2α signaling pathways, have been shown to regulate neuronal differentiation, synaptic plasticity, and neuronal survival. Dysregulation of these pathways has been implicated in abnormal brain development and circuit formation, suggesting that multiple ER stress–related signaling cascades may cooperatively contribute to ASD pathophysiology. However, no significant changes in the expression of synaptic organizers were observed in the striatum following treatment with VPA and 4-PBA. This may be attributed to the postnatal development of the mouse hypothalamus; thus, the effects of fetal administration of 4-PBA on synaptic organizers could not be confirmed [34]. Additionally, the striatum develops later in life; therefore, molecules involved in synaptogenesis may not be affected in this experimental system [34]. Nevertheless, the striatum is a brain region rich in dopamine receptors, and reductions in dopamine receptor signaling are thought to contribute to ASD pathogenesis [35]. Furthermore, the hypothalamus produces oxytocin, which is considered beneficial in ASD treatment [36]. Thus, as both the striatum and hypothalamus are brain regions deeply involved in ASD, further postnatal analyses of ER stress and synaptic regulation will be necessary.

Excessive ER stress during neurodevelopment leads to sustained upregulation of GRP94, reflecting an increased burden of misfolded or aggregation-prone proteins within the endoplasmic reticulum. This GRP94 upregulation is expected to compromise ER quality control for synaptic organizers and cell adhesion molecules. Synaptic organizers such as Shank3 and CADM1 are large, multidomain proteins that require strict ER quality control for proper folding and stability. Under conditions of chronic ER stress accompanied by GRP94 upregulation, these proteins are vulnerable to misfolding and preferential degradation, resulting in their downregulation in ASD models. The protein expression of Shank3 and CADM1 was ameliorated by 4-PBA, despite no changes in the expression of PSD-95, a scaffold protein for NMDA receptors, or NLGN1, which forms excitatory synapses with PSD-95 in the cerebral cortex. Shank3 plays a critical role in synaptogenesis as a glutamate receptor scaffold protein and is highly ubiquitinated in the postsynaptic region, where its proteasomal degradation is regulated in a synaptic activity–dependent manner [37],[38]. The Shank family promotes axon elongation and postsynaptic scaffold organization [39]. Mutations in CADM1 also inhibit dendrite formation and are associated with ASD [40]. These findings suggest that ER stress induced by dysfunctional Shank3 and CADM1 contributes to ASD pathogenesis. In the present study, the suppressed expression of Shank3 and CADM1 observed in the ASD model was restored by 4-PBA treatment, suggesting that the protein-folding repair activity of 4-PBA may reduce ER stress associated with these synaptic organizers. Consequently, we propose that 4-PBA ameliorates ASD-like behaviors by breaking a pathological cycle in which ER stress–induced GRP94 upregulation destabilizes synaptic organizers such as Shank3 and CADM1, through alleviation of ER stress and restoration of ER proteostasis. Although there are many reports on Shank3 in ASD pathogenesis, relatively few studies have examined the mechanistic contribution of CADM1, which participates in signal transduction through metalloproteinase-mediated pathways [41]. While further investigation is required to clarify the relationship between CADM1 and ASD, our findings suggest that CADM1 may contribute to ASD-like symptom development and that its expression can be modulated by 4-PBA. Therefore, in this study, we tested the hypothesis that prenatal alleviation of ER stress by 4-PBA during mid-gestation can prevent the development of ASD-like phenotypes in distinct mouse models.

BTBR mice are widely used as an idiopathic ASD model and have recently been redefined as a neurodevelopmental model characterized by multilevel abnormalities spanning gene regulation, synaptic organization, neural circuit formation, and behavior [42]. In particular, integrative analyses have highlighted disruptions in synaptic organization and cell–cell adhesion, as well as atypical development of cortico-striatal circuits underlying social behavior [42]. These features position BTBR mice as a suitable model for investigating how developmental molecular disturbances translate into persistent social deficits in ASD. To confirm the usefulness of 4-PBA, we evaluated sociability and social novelty using the three-chamber test in this model. We found that prenatal 4-PBA pretreatment selectively reduced GRP94 expression in BTBR mice and ameliorated deficits in sociability to levels comparable to those of control mice. In contrast, 4-PBA did not significantly improve social novelty preference, although a trend toward improvement was observed. These results suggest that abnormal expression of synaptic organizers induced by developmental ER stress is closely associated with social deficits in ASD. Although no significant differences were observed among groups in total measurement time, total distance traveled, or immobility duration, the range of movement was markedly reduced in 4-PBA-treated BTBR mice (Figure 7A). This reduction may be attributable to decreased movement distance accompanied by increased immobility time. However, neither increased immobility nor reduced movement distance was observed in 4-PBA-treated control mice. These changes may reflect multiple factors, including altered cognitive processing, increased attention, or enhanced fear responses; however, direct evidence is currently lacking, and further studies are required.

Finally, it should be noted that 4-PBA possesses dual biological activities as both a chemical chaperone that alleviates ER stress and an HDAC inhibitor. Because the present study did not experimentally distinguish between these mechanisms, the relative contribution of ER stress reduction versus epigenetic modulation to the observed behavioral rescue cannot be fully determined. Nevertheless, given that both VPA and 4-PBA act as HDAC inhibitors, it is unlikely that the amelioration of ASD-like phenotypes by 4-PBA is mediated primarily through HDAC inhibition. Instead, our findings support the interpretation that protein aggregation inhibition and ER stress reduction play major roles in the neuroprotective effects of prenatal 4-PBA treatment.

## Use of AI tools declaration

The authors declare they have not used Artificial Intelligence (AI) tools in the creation of this article.
